# INTEGRATIVE APPROACHES TO MANAGING PAIN IN GERIATRIC PATIENTS

**DOI:** 10.1093/geroni/igz038.1493

**Published:** 2019-11-08

**Authors:** David Kilgore

**Affiliations:** University of California Irvine, orange, California, United States

## Abstract

In the United States, over 100 million people have chronic pain, which is associated not only with high care costs, disability and suffering, but more recently, with growing concerns about potential side effects and complications from medications commonly used to treat pain, in particular NSAIDs and opioid therapies. Pain is a multifaceted issue involving nutritional, psychosocial, biochemical, neurological and physical components. Our current medical model typically addresses pain in a unidimensional mode that often only focuses on medications. The last decade has seen advancement in the evidence base behind a number of Integrative therapies used to treat pain, most of which offer a safe side effect profile and can help expand the toolkit of available options to treat this challenging problem in the older patient. This session will briefly review several examples of Integrative therapies for pain, including Acupuncture, Mind Body treatments, Nutrition and selected supplements.

